# Long-Term Enhancement of Brain Function and Cognition Using Cognitive Training and Brain Stimulation

**DOI:** 10.1016/j.cub.2013.04.045

**Published:** 2013-06-03

**Authors:** Albert Snowball, Ilias Tachtsidis, Tudor Popescu, Jacqueline Thompson, Margarete Delazer, Laura Zamarian, Tingting Zhu, Roi Cohen Kadosh

**Affiliations:** 1 Department of Experimental Psychology, University of Oxford, Oxford, OX1 3UD, UK; 2 Department of Medical Physics and Bioengineering, University College London, London, WC1E 6BT, UK; 3 Clinical Department of Neurology, Innsbruck Medical University, Anichstrasse 35, 6020 Innsbruck, Austria

## Abstract

Noninvasive brain stimulation has shown considerable promise for enhancing cognitive functions by the long-term manipulation of neuroplasticity [1–3]. However, the observation of such improvements has been focused at the behavioral level, and enhancements largely restricted to the performance of basic tasks. Here, we investigate whether transcranial random noise stimulation (TRNS) can improve learning and subsequent performance on complex arithmetic tasks. TRNS of the bilateral dorsolateral prefrontal cortex (DLPFC), a key area in arithmetic [4, 5], was uniquely coupled with near-infrared spectroscopy (NIRS) to measure online hemodynamic responses within the prefrontal cortex. Five consecutive days of TRNS-accompanied cognitive training enhanced the speed of both calculation- and memory-recall-based arithmetic learning. These behavioral improvements were associated with defined hemodynamic responses consistent with more efficient neurovascular coupling within the left DLPFC. Testing 6 months after training revealed long-lasting behavioral and physiological modifications in the stimulated group relative to sham controls for trained and nontrained calculation material. These results demonstrate that, depending on the learning regime, TRNS can induce long-term enhancement of cognitive and brain functions. Such findings have significant implications for basic and translational neuroscience, highlighting TRNS as a viable approach to enhancing learning and high-level cognition by the long-term modulation of neuroplasticity.

## Highlights

• Subjects received TRNS of the bilateral DLPFC while undergoing arithmetic training
• TRNS was uniquely coupled with NIRS, an optical brain imaging technique
• TRNS elicited short- and long-term improvements in trained and untrained material
• Hemodynamic responses suggested enhanced neurovascular coupling efficiency

## Results

### Behavioral Effect of TRNS

**Figure 1:**
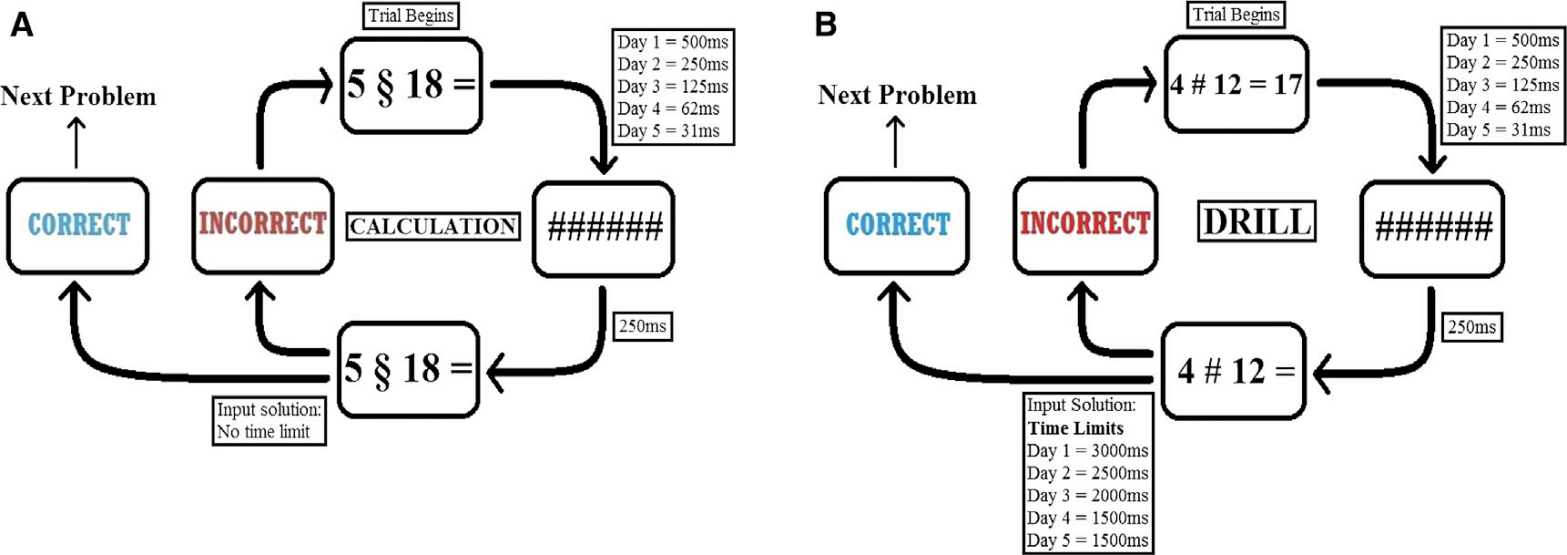
Schematic Representation of the Arithmetic Learning Regimes (A) The calculation task. Answers to each calculation problem were obtained by manipulation of two numerical operands according to a particular algorithm (either [(right number – left number) + 1] + right number or [(right number + left number) − 10] + right number). Participants were instructed to enter their two-digit solution on the number pad of a standard QWERTY keyboard. Positive and negative feedback was provided for each answer (500 ms duration), and participants were only allowed to progress to subsequent problems once they had obtained the correct solution. (B) The drill task. Each trial began with the presentation of two numerical operands accompanied by the problem’s answer. After the initial presentation, the problem would disappear from the screen and reappear without the answer, at which point subjects were required to enter their two-digit solution. Positive and negative feedback was provided (500 ms duration), and if participants answered incorrectly, the whole presentation cycle would repeat. Calculation and drill problems were presented in alternating groups of 18, referred to as “blocks.” In line with previous studies [7], the total number of blocks varied according to the day of training: ten blocks on the first day, 12 on the second, 14 on the third, 16 on the fourth, and 14 on the fifth. The ratio of calculation to drill blocks was the same on each day, at 1:1. For both tasks, the round-edged boxes represent the individual presentation screens, and the square-edged boxes the time delays between each presentation screen.

We assessed two distinct learning regimes: (1) deep-level cognitive processing and (2) shallow-level cognitive processing [6]. In the current context of mathematical cognition, deep- and shallow-level processing have been defined as calculation and drill learning, respectively (Figure 1). Drill learning is characterized by the ability to recall arithmetic “facts” (e.g., 4 × 8 = 32) from memory (rote learning). Extensive repetition of the association between numerical operands and answers is employed as subjects “learn by remembering,” but no knowledge of the arithmetic/mathematical relationship between operands and answers is required [8]. Calculation learning is characterized by the manipulation of numbers according to set procedures or algorithms involving one or several mathematical operations (e.g., 32 − 17 + 5 = 20). It is distinct from drill learning in that an understanding of basic mathematical principles is necessary for effective performance [8]. For each participant, error rates (ERs) and median reaction times (RTs; correct trials only) were calculated for drill and calculation problems presented over the 5 days of training. These data are provided in the Supplemental Information (Figure S1 available online).

#### Assessing Short-Term Neuroplasticity

**Figure 2:**
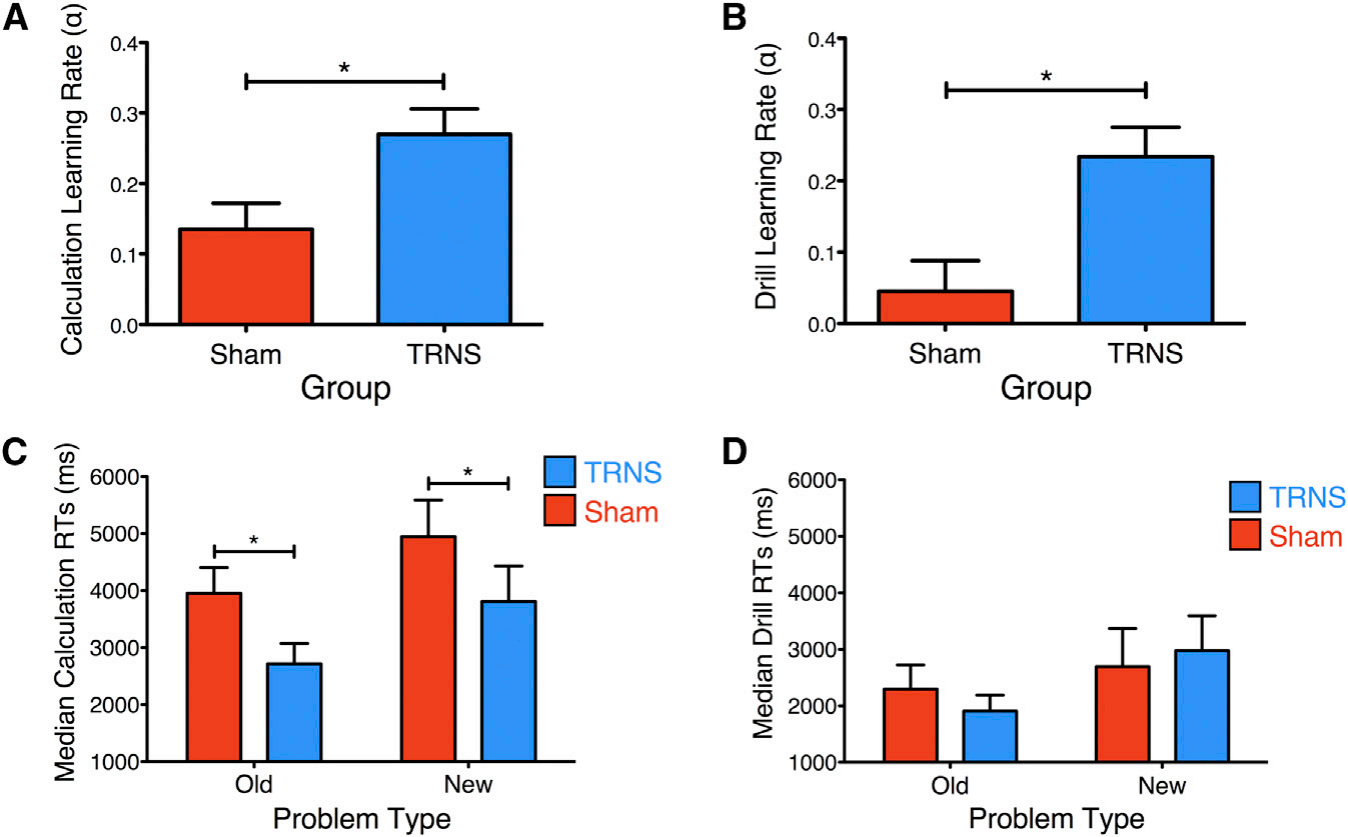
The Effect of TRNS on Arithmetic Performance (A) Calculation learning rates during training were significantly higher in the TRNS group relative to sham controls. (B) Drill learning rates during training were significantly higher in the TRNS group relative to sham controls. (C) Calculation RTs during testing were significantly faster in the TRNS group relative to sham controls for both old and new problems. (D) Drill RTs during testing did not differ between TRNS and sham groups for either old or new problems. Error bars indicate one SEM. Significant differences are marked with asterisks. See also Figure S2.

For assessment of skill acquisition, it is recommended that calculation and drill learning be modeled by fitting of RT data to a power law function [9] (Supplemental Experimental Procedures). This modeling allows one to quantify both initial performance (B) and learning rates (α) for each learning regime. For both calculation and drill arithmetic, there were no significant differences between the groups with regard to initial performance, indicating similar proficiency at the beginning of training (p values > 0.27). In contrast, calculation and drill learning rates were significantly higher for the transcranial random noise stimulation (TRNS) group relative to sham controls [calculation, F(1,22) = 6.75, p = 0.016; drill, F(1,22) = 10.24, p = 0.004, using initial performance as a covariate [10]; Figures 2A and 2B]. This result indicates that TRNS facilitated the speed of learning for both calculation and drill regimes.

Potential confounds arising from our behavioral data were examined, and all further analyses confirmed the robustness of the above findings (Supplemental Results and Figures S1 and S2).

The specificity of the effect of TRNS for arithmetic enhancements was assessed by quantification of the influence of brain stimulation on a selection of cognitive faculties subserved by the dorsolateral prefrontal cortex (DLPFC). Participants’ mental rotation and attentional capacities were tested before and after training. TRNS did not influence performance in these domains, indicating that the effects of stimulation were specific to the trained material, at least in the context of the control tasks employed here (Supplemental Results).

#### Assessing Long-Term Neuroplasticity

For assessment of the long-term effects of cognitive training and TRNS, participants’ performance on old (presented during training) and new (not presented during training) calculation and drill problems was tested 6 months after training. Subjects were contacted without prior notice and asked to solve problems while near-infrared spectroscopy (NIRS) recordings were taken in the absence of further TRNS. No significant interaction existed between calculation problem novelty (old, new) and group (sham, TRNS) (F < 1). However, as predicted on the basis of performance after training, the application of TRNS 6 months prior to testing led to superior long-term performance, compared to sham controls, on old and new calculation problems [RT, t(10) = −1.92, p = 0.04, one-tailed; Figure 2C]. Despite only 12 (six sham, six TRNS) of the original 25 participants being available for recall, these results cannot be explained by biased selection for the testing phase, as there were no significant differences in performance between participants who were available for testing and those who were not (interaction between group and testing availability [yes, no], F < 1, p = 0.43; see also Table S1).

TRNS did not elicit long-term improvements in drill performance: there was no interaction between problem novelty and group (F < 1), and old and new drill problem RTs did not differ significantly between the groups [t(10) = 0.72, p = 0.48; Figure 2D].

Further analysis indicated that the interaction between group and learning regime (calculation or drill) was significant [F(1,10) = 9.31, p = 0.01].

No significant differences existed between the groups when performance, during training or testing, was assessed using ERs (p values > 0.17).

### Physiological Effect of TRNS

NIRS exploits the relative transparency of biological tissue in the NIR region of the electromagnetic spectrum (700–1000 nm) to measure changes in the concentrations of oxyhemoglobin (HbO_2_), deoxyhemoglobin (HHb), and total hemoglobin (HbT), the summation of HbO_2_ and HHb. Functional NIRS describes the measurement of hemodynamic changes specifically associated with brain activation in response to a given stimulus [11].

#### Assessing Short-Term Neuroplasticity

**Figure 3:**
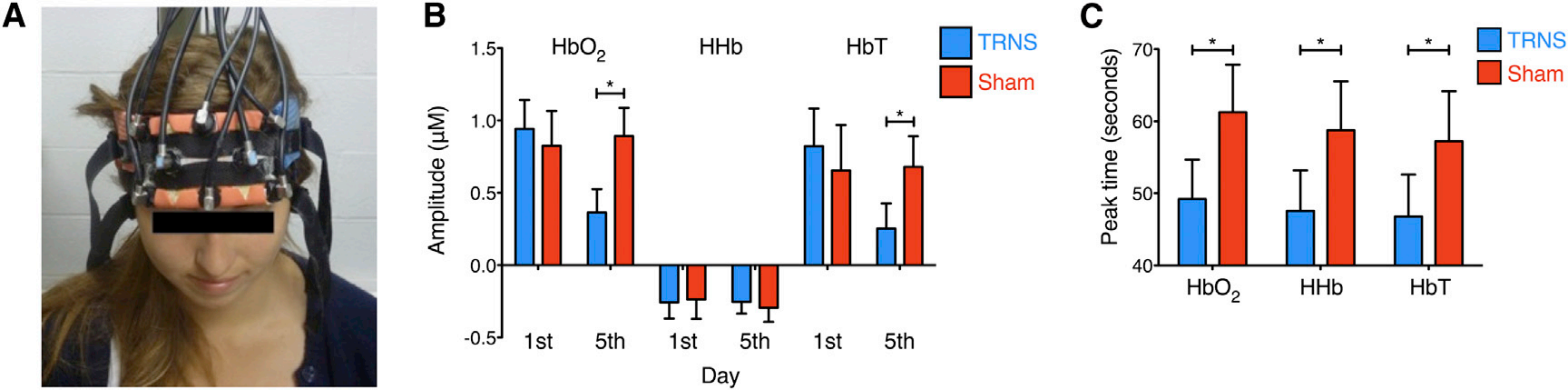
NIRS: The Effect of TRNS on Hemodynamic Response Amplitudes and Latencies within the Left LPFC during Training (A) Combined TRNS-NIRS setup. The NIRS plate (orange) is embedded with two transmitting and six receiving optodes and secured to the forehead: transmitting optodes are capped with blue labels, and receiving optodes are unmarked. Recordings were taken during the training phase on the first day and the fifth (last) day, as well as during the testing phase 6 months later. The fiber optic cables connecting the optodes to the NIRS device can be seen emanating from the top of the image. TRNS electrodes are positioned over the bilateral DLPFC and encased in blue and red saline-soaked sponges, as shown. This innovative TES-NIRS combination allowed us to quantify the hemodynamic response to functional activation and to assess how it varied as a function of brain stimulation and arithmetic training. (B) TRNS reduced the amplitude of HbO_2_ and HbT responses by the end of training. A significant three-way interaction between hemodynamic measure, day, and group in the left LPFC indicates a significant decrease in peak amplitude for HbO_2_ and HbT at the end of the training (fifth day) in the TRNS group relative to sham controls. (C) Reduced peak latencies emerged in the TRNS group compared to sham controls, for HbO_2_, HHb, and HbT responses, independent of day. Both these effects were restricted to the left LPFC. Error bars indicate one SEM. Significant differences are marked with asterisks. See also Figure S3.

A cushioned plate embedded with six receiving and two transmitting optodes was placed on the scalp of each participant to extract NIRS data from the prefrontal cortex (PFC) on the first and last days of training (Figure 3A). In order to differentiate meaningful hemodynamic responses from type I error [12], we first identified the NIRS recording channels that displayed functional activation during cognitive training. Functional activation is characterized by a significant increase in the concentrations of HbO_2_ and HbT, coupled with a decrease in the HHb concentration [13]. In line with previous functional magnetic resonance imaging studies (for a meta-analysis, see 5]), greater arithmetic-induced functional activation was observed for channels over the lateral PFC (LPFC) than the medial PFC [F(1,24) = 6.26, p = 0.02]. Subsequent analysis was therefore focused on the amplitude and latency of peak changes in HbO_2_, HHb, and HbT concentrations within the LPFC that occurred as a function of TRNS. These parameters will henceforth be referred to as “peak amplitude” and “peak latency,” respectively.

A mixed-model analysis of variance was run with hemodynamic measure (HbO_2_, HHb, HbT peak amplitude/latency), learning regime, and day of training (first day, last day) as within-subject factors and group as a between-subject factor. Note that this analysis is completely independent of that used to define activated channels, and thus circular inference is avoided [14].

The interaction between hemodynamic measure (peak amplitude), day, and group was significant [F(2,40) = 4.22, p = 0.02; Figure 3B]. This interaction indicated similar peak amplitudes in both groups at the beginning of training [first day, group X hemodynamic measure interaction, F(2,40) = 0.37, p = 0.69] that evolved into reduced peak amplitudes for HbO_2_ and HbT in the TRNS group relative to sham controls by the end of training [fifth day, group X hemodynamic measure interaction, F(2,40) = 6.59, p = 0.003; due to group effects on the last day for HbO_2_, F(1,20) = 8.64, p = 0.008; HbT, F(1,20) = 4.89, p = 0.04; but not HHb, F(1,20) = 0.21, p = 0.65]. A main effect of group was also found for peak latency, indicating a decrease in peak latency in the TRNS group compared to sham controls [F(1,20) = 6.67, p = 0.02, across HbO_2_, HHb, and HbT; Figure 3C]. Notably, these hemodynamic response effects were specific to the left LPFC and were not observed in the right LPFC (p values > 0.2; Figure S3; Supplemental Discussion).

#### Assessing Long-Term Neuroplasticity

**Figure 4:**
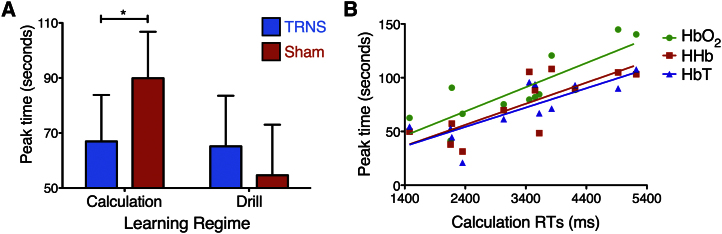
The Effect of TRNS on Hemodynamic Response Latencies during Testing: Relationship with Behavioral Performance (A) A significant two-way interaction existed between learning regime and group for peak latency in the left LPFC, 6 months after the end of training. While the TRNS and sham groups did not differ for drill problems, the TRNS group showed a significant decrease in peak latency compared to sham controls for calculation problems. Error bars indicate one SEM. Significant differences are marked with asterisks. (B) Significant correlations existed between calculation RTs and the peak time of changes in HbO_2_, HHb, and HbT concentrations 6 months after the completion of training. See also Figure S4.

Peak amplitude/latency hemodynamic responses were also assessed during testing 6 months later. The only significant effect was a learning regime X group interaction for peak latency in the left LPFC [F(1,10) = 5.22, p = 0.04; Figure 4A], a finding that mirrors the behavioral results after 6 months. For calculation problems, the TRNS group showed a significant decrease in peak latency compared to sham controls [t(10) = −3.4, p = 0.007]. The groups did not differ for drill problems [t(10) = 0.7, p = 0.5].

To investigate the relationship between our observed behavioral and physiological responses, we correlated calculation RTs with changes in peak latency during testing. The results highlighted significant correlations between the physiological and behavioral parameters (across HbO_2_, HHb, and HbT, r = 0.89, p = 0.00009; HbO_2_, r = 0.83, p = 0.0007; HHb, r = 0.78, p = 0.002; HbT, r = 0.8, p = 0.001; Figures 4B and S4A). Notably, no significant correlations existed when similar analyses assessed the relationship between behavioral performance and peak latency differences on the last day of training (across HbO_2_, HHb, and HbT, r = 0.12, p = 0.7; HbO_2_, r = 0.2, p = 0.52; HHb, r = 0.08, p = 0.8; HbT, r = 0.07, p = 0.82; Figure S4B). The physiological-behavioral correlations with hemodynamic data extracted on the last day of training and those extracted after 6 months differed significantly (p values < 0.05).

#### Assessing the Specificity of Brain Stimulation

In order to examine whether the current results were specific to DLPFC stimulation, we performed a control experiment in which TRNS was applied to the bilateral parietal cortex. The results of this control experiment indicated that both the cognitive enhancement and TRNS-induced hemodynamic responses described above were specific for DLPFC stimulation (Supplemental Results).

## Discussion

The results presented here indicate that TRNS of the bilateral DLPFC can enhance learning with respect to high-level cognitive functions, namely algorithmic manipulation and factual recall in mental arithmetic. When this learning is based on deep-level cognitive processing, as is the case for calculation arithmetic, such enhancements are extremely long-lived both behaviorally and physiologically.

Arithmetic struggles are a characteristic feature of developmental dyscalculia, a learning disorder affecting approximately 5%–7% of the population [15]. In addition, they are present in up to 20% of otherwise healthy children and adults [16] and in a large number of individuals suffering from neurodegenerative disease or stroke [17]. Techniques that can assuage the decline in, or even enhance, cognitive learning and processing are thus highly sought after for both educational and therapeutic purposes [18]. The current results support TRNS as a noninvasive cognitive enhancement tool capable of improving learning in one of the most complex human faculties, mental arithmetic.

One key discovery in the current study is that TRNS-induced changes in calculation performance are maintained for at least 6 months after training. This shows that relatively short stimulation sessions of suitable brain areas can induce long-term learning improvements when coupled with an appropriate training regime. The current results also support the use of learning regimes based on deep-level cognitive processing over those involving shallow-level processing, as the former not only results in long-lasting performance improvements, but also generalization to new, unlearned material. Such generalization is rarely observed in cognitive training studies [19, 20], yet together with long-term performance modulation it will be essential if transcranial electrical stimulation (TES) techniques are to successfully progress to the clinic or classrooms. Improvements that manifest only during the period of stimulation and only for learned material, while scientifically interesting, are less useful in an educational or therapeutic context [21].

TRNS did not improve long-term drill performance. The specificity of the long-term effects of TRNS for calculation arithmetic can be explained by the level of cognitive processing involved in the learning [6]. The strength and longevity of memory formation depends on the depth of processing during the encoding stage [22]. Deep-level processing contributes to the generation of elaborate memory traces better integrated with organized knowledge structures. This allows calculation problems to be solved with reconstructive retrieval processes absent from shallower, drill-type processing. The transfer to new problems in the calculation task supports the proposition that deep-level learning processes modify underlying cognitive systems [22], which are further influenced by concurrent TRNS (Supplemental Discussion).

It can be argued that superior calculation performance in the TRNS group during testing arises not from long-term enhancement of arithmetic abilities per se, but other cognitive processes associated with DLPFC function. While the failure to observe similar long-term effects in the drill condition excludes some possibilities such as long-term memory enhancement, mental arithmetic is a complex faculty based on a variety of cognitive abilities [23]. As such, the current enhancement might stem from the “boosting” of more general DLPFC-associated cognition that is not necessarily specific to arithmetic.

TRNS modulated both the peak amplitude and peak latency of hemodynamic responses to functional activation. At the end of training, the peak amplitudes of HbO_2_ and HbT concentrations in the left LPFC were smaller in the TRNS group than in sham controls. Changes in local HbO_2_ and HbT concentrations are representative of alterations in regional cerebral blood flow (rCBF) and oxygen delivery [24–26], while changes in the local HHb concentration, responses for which were not modified by TRNS, are more sensitive to alterations in the regional cerebral metabolic rate of oxygen consumption (rCMRO_2_) [27]. Our results suggest, therefore, that TRNS elicited changes in corticoexcitability within the left LPFC that significantly reduced rCBF without affecting rCMRO_2_. TRNS, via its amplification of subthreshold oscillatory activity by stochastic resonance, may increase neural firing synchronization within stimulated regions [28]. This could reduce the amount of endogenous electrical noise within such areas, meaning that smaller rCBF responses are required to maintain neural activity. The absence of alterations in rCMRO_2_ with significant changes in neural excitability is well described in the literature [29, 30] (Supplemental Discussion).

That identical metabolic demands (compared to sham controls) were supported by smaller rCBF responses is consistent with a TRNS-induced enhancement of neurovascular coupling efficiency within the left LPFC, a region heavily implicated in arithmetic processing [4, 5]. Peak latency responses further support this proposal. An earlier peak time existed for all three hemoglobin parameters in the TRNS group relative to sham controls on the first and last days of training, and this was maintained, for the calculation task, until the testing phase 6 months later. Previous work has demonstrated that just 4 min of TRNS can modify corticoexcitability [28]. While such rapid modifications would allow TRNS to directly influence peak latency responses in the short-term (training days 1 and 5), long-term (after 6 months) responses occurring in the absence of further stimulation must have arisen via a more indirect mechanism. One possibility is that of structural alterations to the cerebrovasculature. Specific hemodynamic events induced during the training phase could act as precursors to long-term angioplastic modifications. If these were to increase cerebrovascular innervation of neural networks involved in mental arithmetic, faster hemodynamic responses might accompany calculation-induced functional activation, as was observed during the testing phase of the current work. This theory is consistent with animal studies demonstrating significant angiogenesis, and upregulation of the angiogenic vascular endothelial growth factor, after electrical brain stimulation [31].

For the testing phase, we observed strong physiological-behavioral correlations between calculation RTs and peak latencies, which explained up to 79% of the variance. These results serve as good evidence that peak latency responses within the left LPFC are reliable indicators of calculation performance, with earlier peak times indicative of better performance. Notably, despite both superior calculation performance and reduced peak latencies in the TRNS group on day 5 of training, the significant correlations observed after 6 months were not present at this stage. The delayed development of this significant correlation suggests that the behavioral and hemodynamic changes observed on the last day of training are not unrelated, as one might assume [32], but rather act as a scaffold for a more meaningful relationship that manifests in the 6-month training-testing interval.

We have demonstrated that five consecutive days of TRNS-accompanied arithmetic training can markedly improve learning as assessed with both a deep-level cognitive processing calculation task and a shallow-level drill task. Such improvements were accompanied by defined hemodynamic responses consistent with more efficient neurovascular coupling in brain regions associated with mental arithmetic. Both the behavioral and physiological changes displayed extreme longevity, spanning a period of 6 months, but only when learning involved deep-level cognitive processing. By its demonstration of such longevity and, for the calculation task, generalization to new, unlearned material, the present study highlights TRNS as a promising tool for enhancing high-level cognition and facilitating learning. These findings have significant scientific and translational implications for cognitive enhancement in both healthy individuals and patients suffering from disorders characterized by arithmetic deficits [17, 33, 34].

## Experimental Procedures

Detailed experimental procedures are provided in the Supplemental Information.

### Participants

Twenty-five participants were matched for age and gender and randomly assigned to either the TRNS or sham group (TRNS, six males and seven females, mean age = 20.92, SD = 2.10; sham, six males and six females, mean age = 21.42, SD = 3.23). All participants had normal or corrected-to-normal vision and no history of neurological or psychiatric illness. Informed consent was obtained, and volunteers received £60 for their participation. This research was approved by the Berkshire Ethics Committee.

### Arithmetic Tasks: Training

Participants were required to perform two types of learning task: calculation and drill [7, 35]. The tasks are summarized in Figure 1.

### Arithmetic Tasks: Testing

The testing phase included four blocks each of old calculation, new calculation, old drill, and new drill problems. Feedback was not provided, and participants progressed to subsequent problems regardless of whether their previous answer was correct or not.

### Control Tasks

To assess whether TRNS influenced other cognitive domains outside mental arithmetic (perhaps even in a detrimental manner [36]), immediately before (day 1) and after (day 5) training participants completed two control tasks: a mental rotation task and an attention network test (Supplemental Experimental Procedures).

### TRNS

Subjects received TRNS while performing the learning task each day. Two electrodes (5 cm × 5 cm) were positioned over areas of scalp corresponding to the DLPFC (F3 and F4, identified in accordance with the international 10-20 EEG procedure; Figure 3A). Electrodes were encased in saline-soaked synthetic sponges to improve contact with the scalp and avoid skin irritation. Stimulation was delivered by a DC-Stimulator-Plus device (DC-Stimulator-Plus, neuroConn). Noise in the high-frequency band (100–600Hz) was chosen as it elicits greater neural excitation than lower frequency stimulation [37]. For the TRNS group, current was administered for 20 min, with 15 s increasing and decreasing ramps at the beginning and end, respectively, of each session of stimulation. In the sham group current was applied for 30 s after upward ramping and then terminated.

### NIRS

The current study employed a continuous wave (CW) NIRS system (Oxymon MK III, Artinis Medical Systems). This device measures changes in light attenuation at two wavelengths, 764 nm and 858 nm, and utilizes the modified Beer-Lambert law [38] with an age-dependent differential pathlength factor [39] to resolve changes in HbO_2_, HHb, and HbT concentrations within cortical brain tissue.

## Supplemental Information

Document S1. Supplemental Results, Supplemental Discussion, Supplemental Experimental Procedures, Figures S1–S4, and Table S1
