# Supplementary material for: Long-Term Enhancement of Brain Function and Cognition Using Cognitive Training and Brain Stimulation
Source: Curr Biol. 2013 Jun 3;23(11):987–92. doi: 10.1016/j.cub.2013.04.045 (PMC3675670; doi:10.1016/j.cub.2013.04.045)
Supplement: Document S1. Supplemental Results, Supplemental Discussion, Supplemental Experimental Procedures, Figures S1–S4, and Table S1 [file mmc1.pdf]

Current Biology, Volume *23*

Supplemental Information

Long-Term Enhancement of Brain Function and Cognition Using Cognitive Training and Brain Stimulation

Albert Snowball, Ilias Tachtsidis, Tudor Popescu, Jacqueline Thompson, Margarete Delazer, Laura Zamarian, Tingting Zhu, and Roi Cohen Kadosh

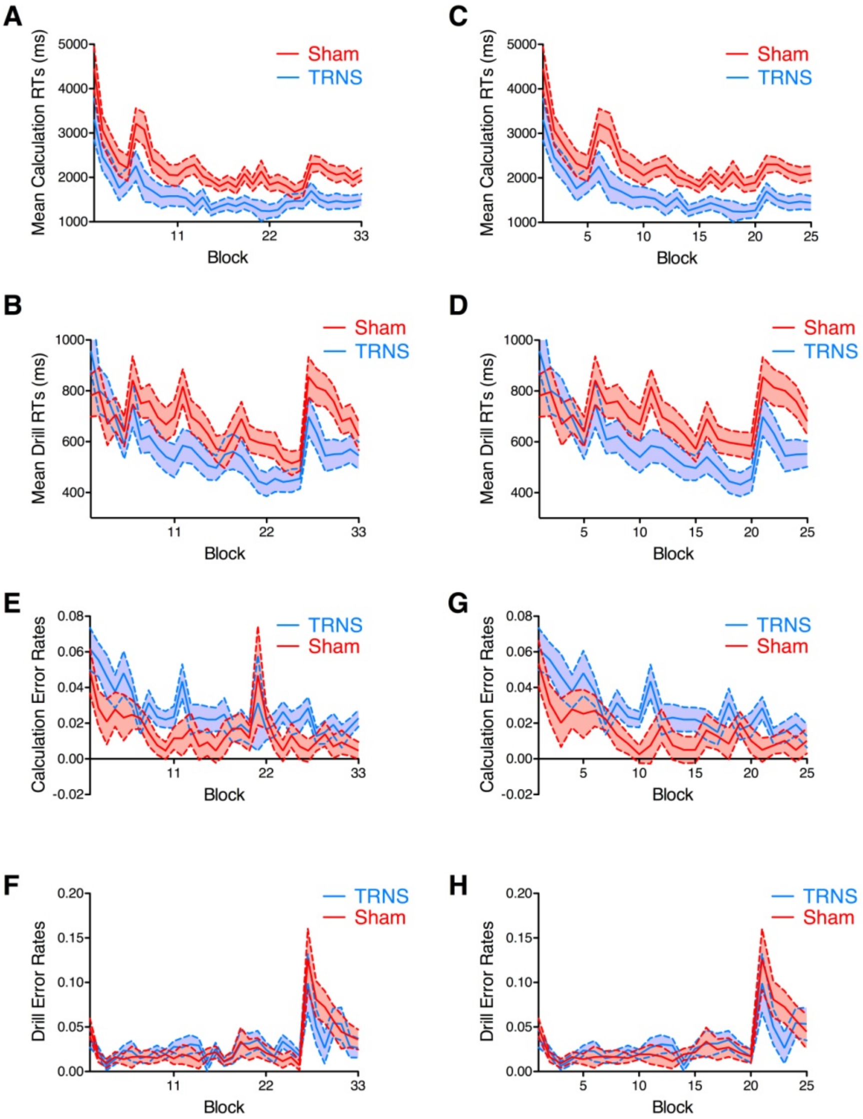

Figure S1. RT and ER data from the training phase, Related to the Results
(A-D) RTs for arithmetic training blocks performed by TRNS and sham subjects. The four panels represent mean RTs for all training blocks (calculation: **A**; drill: **B**), and just the first 5

blocks from each day of training (calculation: **C**; drill: **D**). **(E-H)** ERs for arithmetic training blocks performed by TRNS and sham subjects. The four panels represent ERs for all training blocks (calculation: **E**; drill: **F**), and just the first 5 blocks from each day of training (calculation: **G**; drill: **H**). Data are presented as means (solid lines) ± the standard error of the mean (SEM; dotted lines).

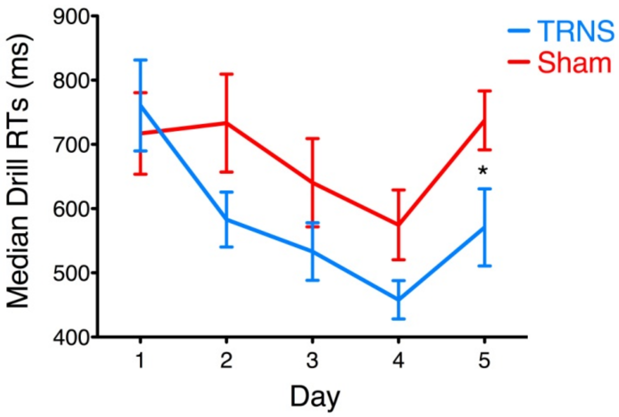

Figure S2. The effect of TRNS on drill performance during training, Related to Figure 2
A significant two-way interaction between day and group for drill RTs indicates a significant difference between the TRNS group and the sham group on the last day of training, representative of better drill performance in stimulated individuals. Given that the presentation time of drill problems was approximately halved each day (from 500ms on day 1 to 31ms on day 5, to keep the task challenging), this improvement occurred in the presence of an increasing cognitive load. Error bars indicate one SEM. Significant differences are marked with asterisks.

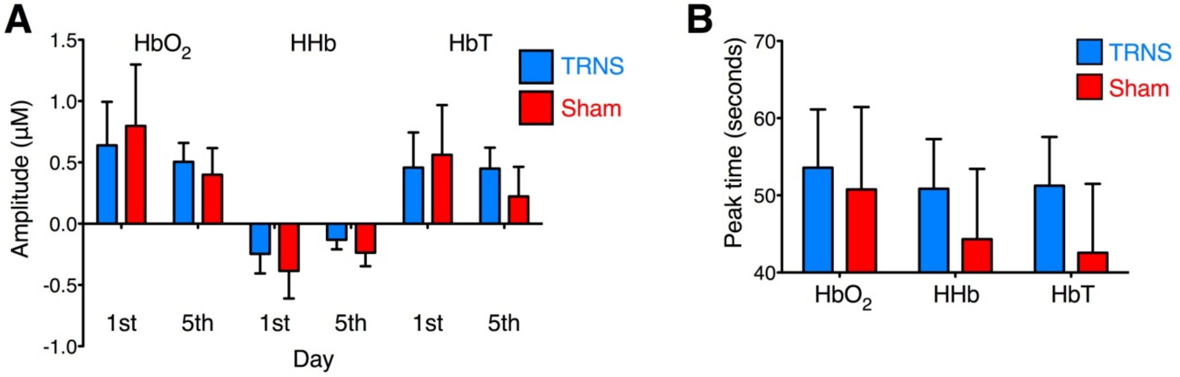

Figure S3. The effect of TRNS on haemodynamic response amplitudes and latencies within the right LPFC during training, Related to Figure 3
(A) In contrast to the left LPFC, the three-way interaction between haemodynamic measure (peak amplitude), day, and group in the right LPFC was not significant. **(B)** For peak latency in the right LPFC neither the main effect of group nor any interaction involving group was significant. Error bars indicate one SEM.

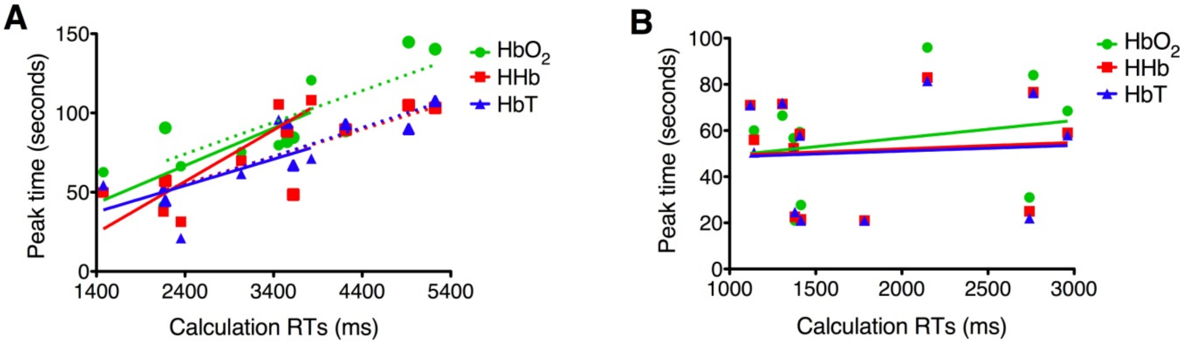

Figure S4. Correlating behavioural performance and haemodynamic physiology, Related to Figure 4
(A) Significant correlations existed between calculation RTs and the peak time of changes in HbO_2, HHb, and HbT concentrations 6 months after the completion of training. Regression lines for sham and TRNS groups are represented by dotted and solid lines, respectively. **(B)** No significant correlations existed between calculation RTs and the peak time of HbO_2, HHb, and HbT concentration changes on the last day of training.

Table S1. Behavioural and haemodynamic effects reported in the main manuscript for the subset of participants that took part in the testing phase after 6 months, Related to the Results

Dependent Variable	Effect	TRNS Group	Sham Group	Inferential Statistics
Drill Learning Rate	Main effect of group	0.18 (0.01)	-0.005 (0.01)	$F(1,9)=63.42$, $p=0.00002$
Calculation Learning Rate	Main effect of group	0.31 (0.05)	0.14 (0.05)	$F(1,9)=4.6$, $p=0.06$
Peak Latency (seconds)	Main effect of group	47.8 (2.22)	63.1 (2.72)	$F(1,8)=18.82$, $p=0.002$
Peak Amplitude (μM)	Interaction between peak amplitude and group	–	–	$F(2,20)=3.42$, $p=0.05$

Supplemental Results

Potential Shortcomings of the Power Law with Respect to the Modelling of Drill Learning

The modelling of practice-based learning with the power law function rests on several assumptions, not all of which were met with respect to drill learning. In particular, task difficulty, which should remain constant throughout training for power law modelling to be valid [1], varied according to day (the time allowed to answer decreased in a stepwise manner from day 1-5). In light of this, the robustness of our findings with respect to drill learning rates was tested with further analysis. Drill RTs were entered into a two-way, repeated measures analysis of variance (ANOVA), with day (days 1-5) as a within-subject factor and group as a between-subject factor. The interaction between day and group was significant ($F(4,92)=2.65$, $p=0.038$), and was due to significantly faster performance in the TRNS group relative to sham controls on day 5 ($t(23)=2.18$, $p=0.04$; **Figure S2**). Together with our learning rate data these results support an enhancement of drill performance by brain stimulation.

Block Number Confounds

The current experimental protocol closely follows previous research [2, 3] in which the number of arithmetic training blocks varied according to day (day 1 to day 5: 10 blocks, 12 blocks, 14 blocks, 16 blocks, 14 blocks). This raises the question of whether RTs varied simply as a function of the number of blocks administered each day (with more blocks-per-day leading to faster RTs), rather than the 'arithmetic learning' suggested. To address this potential confound, our behavioural analysis was modified to include only the first 10 blocks performed on each day of training (5 blocks for drill learning, and 5 blocks for calculation learning, counterbalanced by subject and session; see also **Figure S1**). The results of this modified analysis did not differ from those reported in the main paper. Namely, calculation and drill learning rates were still significantly higher in the TRNS group relative to sham controls (calculation: $F(1,22)=7.9$, $p=0.01$; drill: $F(1,22)=11.07$, $p=0.003$, using initial performance as a covariate [4]). We also examined the effect of stimulation on calculation and drill RTs and ERs for the first 10 training blocks using a factorial design with factors of day (days 1-5) and block (blocks 1-5). In line with our learning rate analysis we observed a significant 3-way interaction (between group, day and block) for both calculation RTs ($F(16,368)=1.8$, $p=0.03$) and drill RTs ($F(16,368)=1.93$, $p=0.02$). This result indicates the absence of a significant difference between the groups at the beginning of training (all $ps>0.17$), which develops into a clear difference towards the end of training (day 5: calculation: $F(1,23)=9.48$, $p=0.005$; drill: $F(1,23)=4.97$, $p=0.03$).

Cognitive Control Tasks

To assess whether TRNS influenced other cognitive domains outside mental arithmetic (perhaps even in a detrimental manner [5]), immediately before (day 1) and after (day 5) training participants completed two control tasks: a mental rotation task (MRT) and an attention network test (ANT). The order in which the two tasks were performed was counterbalanced between subjects, but kept constant for each participant. Questions in the standard MRT comprise figures redrawn from the original Vandenberg-Kuse set [6]. The task quantifies the cognitive ability to mentally rotate given shapes to determine whether

they match a template shape. The ANT was used to quantify activation of the alerting (vigilance) network, the orientating (selection) network, and the conflicting (executive) network [7], and determine whether such activation was influenced by TRNS.
Our results indicate that TRNS did not influence performance on either the MRT or ANT. The lack of effect on mental rotation performance was demonstrated by a non-significant group X day (before training, after training) interaction ($F(1,23)=0.051$, $p=0.82$). Median RTs on the ANT were used to quantify activation of the 3 attention networks in each participant. These values were each subjected to an ANOVA with group and day as factors. There was no significant difference between the groups for any of the 3 attention networks (Alerting: $F(1,23)=0.001$, $p=0.98$; Orienting: $F(1,23)=3.64$, $p=0.07$ (no differences on day 1: $p>0.77$ or day 5: $p>0.14$); Executive: $F(1,23)=0.28$, $p=0.6$), suggesting that alerting, orienting, and more importantly, executive functions, were not affected by TRNS stimulation.
In sum, performance on the cognitive control tasks did not differ as a function of brain stimulation. This is consistent with previous studies that have pinpointed the behavioural effects of TRNS at several minutes after stimulation onset [8], and thus demonstrated that in order to specifically modify the cognitive faculty of interest, in this case mental arithmetic, it is necessary to influence neural activity a short time prior to TES taking effect by performing the cognitive task itself [i.e., state-dependency 9, 10].
The DLPFC is implicated in a variety of cognitive functions [e.g., 11, 12], any number of which might have been affected by brain stimulation. TRNS did not influence performance on the control tasks used in the current study, yet this result does not rule out the possibility that TRNS modulated other cognitive abilities not tested here. Comprehensive assessment of the potential cognitive 'side-effects' of repeated sessions of TRNS is only possible with a range of control tasks testing a variety of faculties. Practical time limitations mean that such assessment is not feasible in a single study. Future investigations employing different control tasks will be necessary to both validate the specificity demonstrated in the current work, and more importantly ensure that cognitive enhancement of one faculty does not come at the expense of another [5].

Assessing the Specificity of Brain Stimulation

In order to examine if the current behavioural and physiological results were site specific for the DLPFC, we performed a control experiment in which near-infrared spectroscopy (NIRS) recordings were taken from the PFC during TRNS or sham stimulation of the bilateral parietal cortices (P3 and P4, based on the international 10-20 EEG procedure) of 26 subjects ($TRNS_{parietal}$: 7 males and 7 females, mean age=20.8, *SD*=1.2; Sham: 5 males and 7 females, mean age=20.8, *SD*=1.3). The bilateral parietal cortex is a key area in mathematical cognition, but is less involved in arithmetic learning [13-15]. Subject selection criteria, arithmetic training protocols and NIRS recording parameters were identical to those described in the main manuscript, and TRNS was the same except for the brain regions stimulated. Note that this control experiment was performed by a different researcher to our primary experiment, and at a later time to assess the specificity of our findings.
Behavioural Effect of $TRNS_{parietal}$. In the case of RTs, calculation learning rates did not differ between $TRNS_{parietal}$ and sham groups ($F(1,23)=0.08$, $p=0.77$, using initial performance as a covariate [4]). Likewise, there was no significant difference between $TRNS_{parietal}$ and sham groups with respect to drill learning rates ($F(1,23)=0.59$, $p=0.45$, using initial performance

as a covariate [4]). With regard to the ANOVA used to analyse drill problem performance, in contrast to the primary experiment, the interaction between day and group was not significant ($F(4,96)=0.36$, $p=0.84$).

No significant effects were observed when ERs were used to assess performance. These results indicate that TRNS of the bilateral parietal cortex did not improve performance on drill or calculation problems over the course of training.

Physiological Effect of $TRNS_{parietal}$. As in our primary experiment, to provide a more refined characterisation of the effect of $TRNS_{parietal}$ on cortical haemodynamic variables such as the amplitude and latency of peak changes in HbO_2, HHb, and HbT concentrations, we entered these parameters into a mixed-model ANOVA with haemodynamic measure (HbO_2, HHb, HbT peak amplitude/latency), learning regime (calculation, drill), and day of training (1st day, 5th day) as within-subject factors, and group ($TRNS_{parietal}$, sham) as a between-subject factor. No significant main effect of group or interaction effect involving group was observed for peak amplitudes or peak latencies in either the left or right LPFC. Specifically, the significant interaction between haemodynamic measure (peak amplitude), day, and group observed in the primary experiment was not significant ($p=0.14$). Likewise, the significant main effect of group for HbO_2, HHb and HbT peak latencies, again observed in the primary experiment, proved non-significant ($p=0.29$).

These results mirror the $TRNS_{parietal}$ behavioural findings, and together they indicate that the behavioural performance improvements observed in the primary experiment, and their associated haemodynamic responses, were specific for TRNS of the DLPFC.

Supplemental Discussion

Quantitative Assessment of Performance

Both RT and accuracy data can be used to gauge arithmetic task performance, yet selecting the measure that provides optimal assessment is often difficult. Complicating the issue is the possible existence of a RT-accuracy trade-off. A participant focus on task accuracy will result in RTs providing the best measure of performance, and vice versa [16].

In the current study, although subjects were instructed to answer problems both as quickly and as accurately as possible, we believe a focus was placed on task accuracy for two reasons. One, during the training phase participants received constant accuracy feedback (by the display of a 'correct' or 'incorrect' message), yet no such feedback was available for RTs. Two, because correct answers were required before progression to subsequent problems, participants likely focussed on high accuracy in order to achieve overall task completion. This accuracy focus explains our observed consistent effects when RT data was used to assess task performance.

Specificity of Haemodynamic Responses for the Left LPFC

One recurring observation in our NIRS analysis was the specificity of significant interactions for the left LPFC, despite bilateral TRNS of the DLPFC. While the bilateral LPFC is involved in arithmetic processing [17], such hemispheric bias might arise from the preferential localisation of 'arithmetic networks' within the left LPFC over the right, especially during arithmetic learning [13]. It has been suggested that TRNS only enhances excitability in neural populations in which there is already a degree of subthreshold activity [8, 18]; in this case those involved in arithmetic task performance. If 'arithmetic networks' have preferential distribution within the left LPFC, the corticoexcitability-enhancing effects of TRNS will be concentrated within this region. Thus increases in neuronal activity will be more likely in the left LPFC than the right, leading to greater TRNS-induced modulation of haemodynamic responses in the former.

We were not able to directly compare the left and right LPFC because for certain conditions some subjects failed to show any significant functional activation in the right LPFC. As such, the inclusion of left and right LPFC data in the same factorial ANOVA would have left us with just 11 subjects. The unwarranted exclusion of many participants that did display significant functional activation within the left LPFC would have led to a severely underpowered design. The relative lack of functional activation in the right LPFC (11 out of 25 subjects, compared to 21 out of 25 subjects in the left LPFC ($\chi^2(1)=7.07$, $p<0.007$)) supports the view that the arithmetic faculties assessed here are processed preferentially within the left LPFC.

Specificity of Long-term Effects for Calculation Arithmetic

Six months after training, during the testing phase, old and new drill problem RTs did not differ significantly between the two stimulation groups (all $ps>0.2$), indicating that TRNS did not induce long-term changes in drill performance. The specificity of the long-term effects of TRNS for calculation problems can be explained by the level of cognitive processing involved in the learning [19], as discussed in the main text. An alternative explanation may lie in the degree of exposure to task-type arithmetic challenges during the training-testing interval. Due to the very specific and unique relationship between numerical operands and answers

in drill problems, subjects were unlikely to have encountered drill-type problems in their everyday lives before testing commenced. In contrast, the calculation task comprised algorithms containing the commonly used arithmetic operations of subtraction and addition. As such, it is highly likely that participants did encounter calculation-type problems in their everyday lives after the conclusion of training. Thus, the lack of maintained performance improvements in the drill task over 6 months might have occurred due to the degeneration of drill-type cognitive processing capacities, which arises from an absence of continued ‘practice’ on drill-type problems. Conversely, calculation performance improvements might have experienced such longevity because of more regular ‘practice’ on algorithm-comprising operations during the 6-month training-testing interval. If this were the case however, one would expect no difference in the processing time between old and new calculation problems. The significant difference we observed between old and new calculation problem RTs ($p<0.05$) supports an explanation for the calculation-specificity of the long-term effects of TRNS that is based on the level of cognitive processing involved in the learning.

Putative Physiological Mechanisms

Our peak amplitude haemodynamic results demonstrated that TRNS elicited changes in corticoexcitability within the left LPFC that significantly reduced regional cerebral blood flow (rCBF) responses without affecting the regional cerebral metabolic rate of oxygen consumption ($rCMRO_2$).

It has been suggested that TRNS, via its amplification of subthreshold oscillatory activity by stochastic resonance, might increase neural firing synchronization within stimulated regions [8]. This could reduce the amount of endogenous electrical noise within such areas, meaning smaller rCBF responses are required to maintain levels of neural activity. Alternatively, TRNS might elicit downstream increases in baseline cortical blood flow. If HbO_2 and HbT concentration increases during functional activation are physiologically restricted to a particular maximum, then the smaller rises in the TRNS group relative to sham controls may occur simply because, in the stimulated group, there is less ‘room’ for concentration increases given the higher resting baseline values of cortical blood flow. Unfortunately, validation of such a proposal requires measurement of absolute haemoglobin concentrations not possible with the CW NIRS device used in the current study.

The absence of alterations in $rCMRO_2$ with significant changes in neural activity is well described in the literature [20, 21]. For example, $CMRO_2$ increases linearly with increasing synaptic activity, but only beyond a certain threshold, an observation consistent with the presence of a tissue oxygen buffer [21-23] or the up-regulation of non-oxidative metabolic processes (e.g. glycolysis) with small increases in synaptic activity [21, 24, 25].

Supplemental Experimental Procedures

Participants

29 volunteers (12 male, all right-handed, mean age=21.16, *SD*=2.66) from the University of Oxford took part in the experiment, which spanned a period of 5 consecutive days for each participant. The data from 4 (2 male) subjects were excluded due to dropout. All participants had normal or corrected-to-normal vision, no implanted metal objects, and no history of seizures, or neurological or psychiatric illness. Informed consent was obtained before the start of the experiment, and volunteers received £60 (~£10/hr) for their participation. Subjects were matched for age and gender. From each pair one individual was randomly chosen to receive real TRNS stimulation while the other received sham stimulation (TRNS: 6 males and 7 females, mean age=20.92, *SD*=2.10; Sham: 6 males and 6 females, mean age=21.42, *SD*=3.23). Both the experimenter who assigned participants to the two stimulation groups and the experimenter who administered the stimulation were blinded, and subjects were unaware of the existence of a sham condition. Within the stimulation groups, participants were split into Drill and Calculation divisions, which determined whether they performed drill or calculation problems first, and into subject sets 1-4, which determined the calculation algorithm an individual performed, and the numerical operands provided to solve that algorithm.

Note that the current study did not include a "sham" training condition, which examines the effect of TRNS alone by administering real or sham stimulation in the absence of training. As we were primarily interested in the impact of TRNS on the two distinct learning regimes, the usefulness of a "sham" training condition as a control was questionable, and therefore excluded from our protocol. The results of our control tasks (see '*Cognitive Control Tasks*' above) suggest that TRNS does not modulate the DLPFC in a general fashion, rather its effects are observed for learned material after stimulation has been combined with cognitive training.

Experimental Design: Training Phase

Participants were required to perform two types of learning task: calculation (denoted by the symbol §) and drill (denoted by #). The tasks were derived from those originally developed by Rickard [3] and Delazer et al. [2].

For the calculation task (**Figure 1A**) each problem consisted of two numerical operands (one single-digit, one double-digit), and four different sets of operands were presented to participants in each of the four subject sets. Participants were provided at the start of the experiment with an algorithm with which to solve each calculation equation. The algorithms were as follows:

Algorithm 1: [(Right Number – Left Number) + 1] + Right Number

Algorithm 2: [(Right Number + Left Number) -10] + Right Number

Participants in subject sets 1 and 3 performed Algorithm 1, while those in subject sets 2 and 4 performed Algorithm 2.

Participants were instructed to enter their two-digit solution on the number pad of a standard QWERTY keyboard. Positive and negative feedback was provided for each answer (500ms duration), and subjects were only allowed to progress to the next problem once they had obtained the correct solution. Participants were instructed to perform their respective algorithmic manipulation mentally for every calculation problem encountered, and not attempt to commit answers to memory.

As in the calculation task, each problem in the drill task (**Figure 1B**) consisted of two numerical operands that varied according to subject set. In this task, however, each trial began with the presentation of the two operands accompanied by the problem's answer. As in previous studies [2], the presentation time was reduced on each day of training: 500ms on day 1, 250ms on day 2, 125ms on day 3, 62ms on day 4, and 31ms on day 5. After the initial presentation, the problem would disappear from the screen for 250ms, and reappear without the answer, at which point subjects were required to enter their two-digit solution. Positive and negative feedback was provided (for 500ms), and if participants answered incorrectly, the whole presentation cycle would repeat. For drill problems, subjects were unaware of the algorithm linking operands to answers, and were instructed to retrieve solutions from memory throughout the week.

Calculation and drill problems were presented in alternating groups of 18, which constitute a single 'block' of problems. In line with previous studies [2], the total number of blocks varied according to the day of training: 10 blocks on the 1st day; 12 on the 2nd; 14 on the 3rd; 16 on the 4th; and 14 on the 5th. The ratio of calculation to drill blocks was the same on each day, at 1:1. Participants were instructed to answer each problem as quickly and efficiently as possible, not sacrificing speed for accuracy or vice versa.

Experimental Design: Testing Phase

Participants had not been made aware during training that they would be recalled. The testing phase included 4 blocks each of old calculation, new calculation, old drill and new drill problems. New drill problems were included to ensure participants had not discovered the arithmetic association between operands and answers, and were indeed providing solutions based on memory recall alone. Subjects were allowed to re-familiarise themselves with their respective algorithm before testing commenced. Feedback was not provided and participants progressed to subsequent problems regardless of whether their previous answer was correct or not.

Experimental Design: TRNS Protocol

Subjects received TRNS of the bilateral DLPFC while performing the learning task each day. Two electrodes (each 5cm x 5cm) were positioned over areas of scalp corresponding to the DLPFC (F3 and F4, identified in accordance with the international 10-20 EEG procedure; **Figure 3A**). Electrodes were encased in saline-soaked synthetic sponges to improve contact with the scalp and avoid skin irritation. An electrical stimulation device (DC-Stimulator-Plus, neuroConn, Germany) generated samples at a rate of 1280 per second, and each sample was randomly assigned a current between -500µA and +500µA, with current amplitudes following a normal distribution [18]. The direct current offset was set at 0. Noise in the high frequency band (100-600Hz) was chosen, as frequencies in this range have been shown to elicit greater neural excitation than lower frequency stimulation [18]. For the TRNS group,

current was administered for 20 minutes, with 15-second increasing and decreasing ramps at the beginning and end, respectively, of each session of stimulation. In the sham group current was applied for 30 seconds after upward ramping and then terminated.

While few studies have demonstrated an inability of participants to distinguish between real TRNS and sham stimulation [26], TRNS is known to have a higher cutaneous perception threshold than transcranial direct current stimulation (TDCS), a form of transcranial electrical stimulation (TES) for which several papers have demonstrated the inability of participants to distinguish between real and sham stimulation [26, 27]. Stimulation started at the beginning of every training session, but always finished prior to its completion. Electrodes were left in position until the conclusion of the learning task, and participants were never made aware that the stimulation had ended. Stimulation parameters were set by one experimenter with no day-to-day access to either the participants or the experimenter running the study: the device's display screen was subsequently covered to ensure that the experimenter supervising the participants remained blinded throughout.

TRNS was preferred over other non-invasive brain stimulation techniques for several reasons. First, TRNS has a higher cutaneous perception threshold than other TES methods such as TDCS [26]. The perceptual difference between real and sham stimulation is therefore less extreme, making TRNS easier to blind. Second, TRNS, unlike TDCS, stimulates targeted brain regions in a polarity-independent fashion [18]. This is an important property when the brain region(s) of interest display(s) bilateral involvement in the given cognitive faculty, as is the case for the DLPFC and arithmetic processing investigated in the current study (however, see '*Specificity of Haemodynamic Responses for the Left LPFC*' above). Polarity independence allows one to stimulate both hemispheres simultaneously during training. Moreover, questions such as where to place the reference electrode, which are often a source of contention in TDCS studies, are less relevant for polarity-independent stimulation. And third, TRNS was preferred over transcranial magnetic stimulation (TMS) as application of the latter for 5 consecutive days can increase the likelihood of seizures [28]. TMS is also less 'perceptually comfortable' than TRNS, making it harder to blind for reasons mentioned above. We placed great emphasis on comfort, as we wanted our subjects to be able to focus on the training material with minimal distractions, if any, from the concurrent stimulation.

Experimental Design: NIRS

The current study employed a continuous wave (CW) NIRS system (Oxymon MK III, Artinis Medical Systems BV, The Netherlands). This system measures changes in light attenuation at two wavelengths, 764nm and 858nm, and utilises the modified Beer-Lambert law [29] with an age-dependent differential pathlength factor [30] to resolve changes in HbO_2, HHb and HbT concentrations within cortical brain tissue. The system is comprised of 12 light sources that are pulsed sequentially, based on the time-sequenced principle, and 2 avalanche photodiode detectors. The optode configuration was the 8-channel split, which allows 8 channel recordings with an optode distance of 40mm.

A cushioned plate embedded with the optodes was placed on the forehead of each participant over the PFC (**Figure 3A**). The experiment was performed in a dimly lit room to reduce ambient light levels, which can cause background noise in the signals from the receiving optodes. Recordings were taken during the training phase on the 1st day and the 5th (last) day, as well as during the testing phase 6 months later. To obtain sufficient baseline

values for HbO_2, HHb, and HbT concentrations, the presentation of arithmetic problems during the three imaging sessions was slightly altered: 2-minute blank screens were included at the beginning and end of each session, and 30-second blank screens inserted between successive blocks of problems.

NIRS Analysis. Graphical read-outs of HbO_2, HHb, and HbT concentration changes were generated using Oxysoft computer software (Artinis Medical Systems BV, The Netherlands) at an original sampling rate of 10Hz. These were then exported at 1Hz time intervals with in-house developed software based in MATLAB (MathWorks).

The first level of analysis identified the blocks and channels that demonstrated functional activation. This approach is highly iterative and aims to reduce the classification of false positives with functional NIRS as reported in recent literature [31]. The time series data of HbO_2, HHb and HbT concentration changes were first detrended to remove drift introduced both by the system and any slowly changing unrelated physiological signals. Individual blocks were then tested for the presence of a haemodynamic response consistent with functional activation. The baseline window was defined as the 20 seconds immediately prior to the onset of each block of problems, and 20-second activation windows were then defined separately for HbO_2, HHb and HbT, for each block independently. This was achieved through the use of an automated algorithm that scanned the HbO_2 and HbT signals following the beginning of each block to identify the 20-second window of maximal concentration increase, and then the overlapping HHb window to identify the maximal concentration decrease in this parameter. Significant changes in the concentrations of HbO_2, HbT and HHb were identified using a paired t-test: blocks that met the haemodynamic criteria detailed above ($p<0.05$ for [HbO2], [HbT]>0, and $p<0.05$ for [HHb]<0) were retained for further analysis.

The Power Law Function

To assess skill acquisition, it is recommended in the case of RTs that calculation and drill learning be modelled by fitting the data to the following power law function [1]:

$$RT = B(N)^{-\alpha}$$

Where N represents the number of the given arithmetic training block, B the RT on the first block (N=1), and α the gradient of the power law curve (i.e. the learning rate).

The power law of practice states that performance increases resulting from practice follow a negatively accelerating power function; that is, practice is associated with improvements in performance that are initially large, yet decrease in magnitude with an increasing trial number [1]. The power law is used to model practice-based learning on a wide variety of task types, including mental arithmetic [1]. Indeed studies investigating the effect of practice on arithmetic abilities have confirmed a good fit of the power function to both calculation and drill learning [3, 32].

Supplemental References

1. Newell, A., and Rosenbloom, P. (1981). Mechanisms of skill acqusition and the law of practice. In Cognitive skills and their acquisition, J.R. Anderson, ed. (Hillsdale, NJ: Erlbaum), pp. 1-55.
2. Delazer, M., Ischebeck, A., Domahs, F., Zamarian, L., Koppelstaetter, F., Siedentopf, C.M., Kaufmann, L., Benke, T., and Felber, S. (2005). Learning by strategies and learning by drill—evidence from an fMRI study. NeuroImage *25*, 838-849.
3. Rickard, T.C. (1997). Bending the power law: A CMPL theory of strategy shifts and the automatization of cognitive skills. J. Exp. Psychol. Gen. *126*, 288-311.
4. Jolles, D., and Crone, E.A. (2012). Training the developing brain: a neurocognitive perspective. Front. Hum. Neurosci. *6*.
5. Iuculano, T., and Cohen Kadosh, R. (2013). The mental cost of cognitive enhancement. J. Neurosci. *33*, 4482-4486.
6. Vandenberg, S.G., and Kuse, A.R. (1978). Mental rotations, a group test of three-dimensional spatial visualization. Percept. Motor Skil. *47*, 599-604.
7. Fan, J., McCandliss, B.D., Sommer, T., Raz, A., and Posner, M.I. (2002). Testing the efficiency and independence of attentional networks. J. Cogn. Neurosci. *14*, 340-347.
8. Chaieb, L., Kovacs, G., Cziraki, C., Greenlee, M., Paulus, W., and Antal, A. (2009). Short-duration transcranial random noise stimulation induces blood oxygenation level dependent response attenuation in the human motor cortex. Exp. Brain Res. *198*, 439-444.
9. Cohen Kadosh, R., Soskic, S., Iuculano, T., Kanai, R., and Walsh, V. (2010). Modulating neuronal activity produces specific and long lasting changes in numerical competence. Curr. Biol. *20*, 2016-2020.
10. Silvanto, J., Muggleton, N., and Walsh, V. (2008). State dependency in brain stimulation studies of perception and cognition. Trends Cogn. Sci. *12*, 447-454.
11. Duncan, J. (2001). An adaptive coding model of neural function in prefrontal cortex. Nat. Rev. Neurosci. *2*, 820-829.
12. Duncan, J., and Owen, A.M. (2000). Common regions of the human frontal lobe recruited by diverse cognitive demands. Trends Neurosci. *23*, 475-483.
13. Zamarian, L., Ischebeck, A., and Delazer, M. (2009). Neuroscience of learning arithmetic—Evidence from brain imaging studies. Neurosci. Biobehav. Rev. *33*, 909-925.
14. Butterworth, B., and Walsh, V. (2011). Neural basis of mathematical cognition. Curr. Biol. *21*, R618-R621.
15. Menon, V. (2010). Developmental cognitive neuroscience of arithmetic: implications for learning and education. ZDM *42*, 515-525.
16. Pachella, R. (1974). The interpretation of reaction time in information processing research. In Human information processing: Tutorials in performance and cognition, B.H. Kantowitz, ed. (Hillsdale, NJ: Erlbaum), pp. 41–82.
17. Arsalidou, M., and Taylor, M.J. (2011). Is 2+2=4? Meta-analyses of brain areas needed for numbers and calculations. NeuroImage *54*, 2382-2393.

18. Terney, D., Chaieb, L., Moliadze, V., Antal, A., and Paulus, W. (2008). Increasing human brain excitability by transcranial high-frequency random noise stimulation. J. Neurosci. *28*, 14147-14155.
19. Craik, F.I.M., and Lockhart, R.S. (1972). Levels of processing: A framework for memory research. J. Verb. Learn. Verb. Behav. *11*, 671-684.
20. Uludağ, K., Dubowitz, D.J., Yoder, E.J., Restom, K., Liu, T.T., and Buxton, R.B. (2004). Coupling of cerebral blood flow and oxygen consumption during physiological activation and deactivation measured with fMRI. NeuroImage *23*, 148-155.
21. Sheth, S.A., Nemoto, M., Guiou, M., Walker, M., Pouratian, N., and Toga, A.W. (2004). Linear and Nonlinear Relationships between Neuronal Activity, Oxygen Metabolism, and Hemodynamic Responses. Neuron *42*, 347-355.
22. Buxton, R.B. (2001). The Elusive Initial Dip. NeuroImage *13*, 953-958.
23. Mayhew, J., Johnston, D., Martindale, J., Jones, M., Berwick, J., and Zheng, Y. (2001). Increased Oxygen Consumption Following Activation of Brain: Theoretical Footnotes Using Spectroscopic Data from Barrel Cortex. NeuroImage *13*, 975-987.
24. Fox, P.T., Raichle, M.E., Mintun, M.A., and Dence, C. (1988). Nonoxidative glucose consumption during focal physiologic neural activity. Science *241*, 462-464.
25. Prichard, J., Rothman, D., Novotny, E., Petroff, O., Kuwabara, T., Avison, M., Howseman, A., Hanstock, C., and Shulman, R. (1991). Lactate rise detected by 1H NMR in human visual cortex during physiologic stimulation. Proc. Natl. Acad. Sci. *88*, 5829-5831.
26. Ambrus, G.G., Paulus, W., and Antal, A. (2010). Cutaneous perception thresholds of electrical stimulation methods: Comparison of tDCS and tRNS. Clin. Neurophysiol. *121*, 1908-1914.
27. Gandiga, P.C., Hummel, F.C., and Cohen, L.G. (2006). Transcranial DC stimulation (tDCS): a tool for double-blind sham-controlled clinical studies in brain stimulation. Clin. Neurophysiol. *117*.
28. Rossi, S., Hallett, M., Rossini, P.M., and Pascual-Leone, A. (2009). Safety, ethical considerations, and application guidelines for the use of transcranial magnetic stimulation in clinical practice and research. Clin. Neurophysiol. *120*, 2008-2039.
29. Delpy, D.T., and Cope, M. (1997). Quantification in tissue near–infrared spectroscopy. Philo. Trans. Roy. Soc. Lond. B Biol. Sci. *352*, 649-659.
30. Duncan, A., Meek, J.H., Clemence, M., Elwell, C.E., Tyszczuk, L., Cope, M., and Delpy, D. (1995). Optical pathlength measurements on adult head, calf and forearm and the head of the newborn infant using phase resolved optical spectroscopy. Phys. Med. Biol. *40*, 295.
31. Kirilina, E., Jelzow, A., Heine, A., Niessing, M., Wabnitz, H., Brühl, R., Ittermann, B., Jacobs, A.M., and Tachtsidis, I. (2012). The physiological origin of task-evoked systemic artefacts in functional near infrared spectroscopy. NeuroImage *61*, 70-81.
32. Delaney, P.F., Reder, L.M., Staszewski, J.J., and Ritter, F.E. (1998). The strategy-specific nature of improvement: The Power Law Applies by Strategy Within Task. Psychol. Sci. *9*, 1-7.
